# Influence of growth structures and fixed appliances on automated cephalometric landmark recognition with a customized convolutional neural network

**DOI:** 10.1186/s12903-023-02984-2

**Published:** 2023-05-10

**Authors:** Teodora Popova, Thomas Stocker, Yeganeh Khazaei, Yoana Malenova, Andrea Wichelhaus, Hisham Sabbagh

**Affiliations:** 1 Department of Orthodontics and Dentofacial Orthopedics, University Hospital, LMU Munich, Goethestrasse 70, 80336 Munich, Germany; 2 Department of Statistics, Statistical Consultation Unit, StaBLab, LMU Munich, Akademiestr. 1, 80799 Munich, Germany; 3 Department of Oral and Maxillofacial Surgery, University Hospital, LMU Munich, Lindwurmstrasse 2a, 80337 Munich, Germany

**Keywords:** Cephalometry, Convolutional neural network, Deep learning, Orthodontics, Cephalometric landmarks

## Abstract

**Background:**

One of the main uses of artificial intelligence in the field of orthodontics is automated cephalometric analysis. Aim of the present study was to evaluate whether developmental stages of a dentition, fixed orthodontic appliances or other dental appliances may affect detection of cephalometric landmarks.

**Methods:**

For the purposes of this study a Convolutional Neural Network (CNN) for automated detection of cephalometric landmarks was developed. The model was trained on 430 cephalometric radiographs and its performance was then tested on 460 new radiographs. The accuracy of landmark detection in patients with permanent dentition was compared with that in patients with mixed dentition. Furthermore, the influence of fixed orthodontic appliances and orthodontic brackets and/or bands was investigated only in patients with permanent dentition. A t-test was performed to evaluate the mean radial errors (MREs) against the corresponding SDs for each landmark in the two categories, of which the significance was set at *p* < 0.05.

**Results:**

The study showed significant differences in the recognition accuracy of the Ap-Inferior point and the Is-Superior point between patients with permanent dentition and mixed dentition, and no significant differences in the recognition process between patients without fixed orthodontic appliances and patients with orthodontic brackets and/or bands and other fixed orthodontic appliances.

**Conclusions:**

The results indicated that growth structures and developmental stages of a dentition had an impact on the performance of the customized CNN model by dental cephalometric landmarks. Fixed orthodontic appliances such as brackets, bands, and other fixed orthodontic appliances, had no significant effect on the performance of the CNN model.

**Supplementary Information:**

The online version contains supplementary material available at 10.1186/s12903-023-02984-2.

## Introduction

Cephalometric analysis involves identifying common landmarks, quantifying the various relationships between them, and diagnosing the correlations in a patient's craniofacial morphology. However, during the cephalometric tracing, sources of error or inter-observer variability may lead to low reproducibility of the observations [1–5]. Since the process of manually placing the landmarks in a cephalogram is also time consuming [6, 7], several studies have proposed frameworks using Deep Learning and Convolutional Neural Networks (CNN) for an automatic landmark recognition in lateral cephalometric radiographs [8–20]. One of the first publications about an automatic system for cephalometric landmark detection was published in 1986 [7], describing a knowledge-based line tracker guided by a reference map. Subsequently, an algorithm-based gray-scale mathematical morphology was presented [21]. In 2014-2015, several strategies for cephalometric landmark detection were introduced after a scientific challenge proposal by the International Symposium on Biomedical Imaging (ISBI). The game-theoretic landmark detection and random forest-based shape model [22] and the random forest regression-voting model [23] both performed favorably in the challenge. Recent studies focused on investigating the performance and reliability of different Convolutional Neural Network (CNN) models for cephalometric analysis [10, 15, 24–28]. As automated cephalometric software platforms are now available from different companies (e.g. OneCeph, Hyderabad, India; CellmatIQ, Hamburg, Germany; WebCeph, Republic of Korea; AudaxCeph, Ljubljana, Slovenia) more recent studies have focused on evaluating their accuracy [15, 29–33]. While the benefits of artificial intelligence in recognizing cephalometric landmarks have been acknowledged [34, 35], the need for further research regarding its accuracy in different clinical settings was recognized [36–38]. Previous studies tested the frameworks only on radiographs of patients with permanent dentition [24, 30, 33] or did not mention these characteristic of the datasets at all [15–17, 25, 26]. Despite the promising potential of automatic landmark recognition, conclusions and research regarding some clinical aspects are still lacking. Hence, this study aims to investigate the influence of growth structures, such as tooth germs in mixed dentitions, and fixed appliances on automated cephalometric landmark recognition.

In particular, the null hypothesis that developmental stages of a dentition, fixed orthodontic appliances or other dental appliances do not affect the accuracy of a customized artificial model for automatic detection of cephalometric landmarks shall be tested. For these purposes, a CNN model with commonly used architecture [39] was developed and the overall accuracy of the model and its validity was evaluated. Finally, the CNN was applied to investigate differences between the distinct patient groups.

## Materials and methods

### Study design

This retrospective diagnostic study was approved by the LMU Ethics Commitee (Ref. No 19–863). Cephalometric radiographs were obtained from the archives of the Department of Orthodontics and Dentofacial Orthopedics, University Hospital, LMU Munich. For this study, a Convolutional Neural Network (CNN) was developed for automatic recognition of cephalometric landmarks. The accuracy of landmark recognition in patients with permanent dentition was compared with that of patients with mixed dentition (both groups included radiographs without fixed orthodontic appliances). In addition, this study investigated the influence of fixed orthodontic appliances and orthodontic brackets and/or bands among patients with permanent dentition only. For reporting this study, the guidelines of the Checklist for Artificial Intelligence in Dentistry [40] and the Standards for Reporting of Diagnostic Accuracy Studies (STARD) [41] were followed.

### Data, sampling and references standard

The patient sample was intended to be as comprehensive as possible, therefore exclusion criteria were limited to craniofacial anomalies and to images of poor quality and/or incorrect positioning of the skull, which might affect landmark recognition. Images of growing and adult patients with or without fixed orthodontic appliances, dental restorations and osteosynthesis plates were included. The distribution of data by age, sex and ethnicity are shown in Table 1. All included radiographs were obtained prior to the study from the same X-ray unit (Orthophos, Sirona, Germany) and had an image size of 2020 × 2012 pixels, where one pixel equals to a square with a length of 0.1 mm on each side and an area of 0.01 mm^2^. Out of 1151 images, 251 images were excluded applying the exclusion criteria, 430 were included in the training dataset and 460 images were used as the test dataset.

**Table 1:** Distribution of data by age, sex and ethnicity in the training and test dataset

	**Train Dataset**		**Test Dataset**	
	***n***		***n***	
age < 6	10	2%	3	1%
6 < age < 13	149	35%	233	51%
age > 13	271	63%	224	48%
female	203	47%	250	54%
male	227	53%	210	46%
caucasians	416	97%	452	98%
non-caucasians	14	3%	8	2%

Cephalometric analysis included 16 key landmarks for the orthodontic diagnosis of the skeletal and dental anatomy. Since soft tissue cephalometric landmarks are rarely located in the proximity of developing tooth germs or fixed orthodontic appliances, they were not considered in the present study. The positions of 16 cephalometric reference points (Table 2) were manually identified by two examiners (last year orthodontic residents), who traced a maximum of 10 lateral cephalograms a day. The annotated radiographs were revised by an orthodontic specialist (10 years of experience) who verified a maximum of 5 images daily, discrepancies were then resolved by consensus. The verified dataset was used as a reference for the training, testing and validation of the CNN model. The verified dataset was used as a reference for the training, testing and validation of the CNN model.

**Table 2:** Abbreviations and definitions of the cephalometric landmarks used in the study

**Abbreviation**	**Landmark**	**Definition**
***A-Point***	Subspinale	Most concave point on the anterior contour of the maxillary alveolar process in the midsagittal plane
***Ap1***	Apex superior	Furthest apical point of the upper central incisors
***Ap***$\overline{1 }$	Apex inferior	Furthest apical point of the lower central incisors
***ANS***	Anterior nasal spine	The most anterior point of the anterior nasal spine in the median sagittal plane
***Art***	Articulare	The intersection of the inferior surface of the cranial base and the posterior border of the ascending rami of the mandible
***B-Point***	Supramentale	Most concave point on the anterior contour of the mandibular alveolar process in the midsagittal plane
***Ba***	Basion	Most anterior point on foramen magnum
***Me***	Menton	The lowest point on the mandibular symphysis in the midline
***Is1***	Incision Superior	The tip of the incisal edge of the most labially positioned upper central incisors
***Is***$\overline{1 }$	Incision Inferior	The tip of the incisal edge of the most labially positioned lower central incisors
***N***	Nasion	The most anterior point on frontonasal suture
***Pog***	Pogonion	The most ventral point of the bony chin in the median sagittal plane
***PNS***	Posterior nasal spine	The intersection of a continuation of the anterior wall of the pterygopalatine fossa and the floor of the nose
***S***	Sella	Midpoint of Sella turcica
***T1***	Gonion superiorus	Most posterior point of posterior border of ramus ascendens
***T2***	Gonion inferius	Most inferior point of gonion area

### Training dataset

The training data set consisted of a total of 430 images including patients with both permanent dentition and mixed dentition, as well as radiographs with fixed orthodontic appliances, orthodontic brackets and/or bands, osteosynthesis plates, implants, dental prosthetic restorations and root canal treatments. The images were divided into training images (90%) and validation images (10%). The training images are used to adjust and optimize the model so that the CNN "learns" how to perform its task, while the validation images provide an objective evaluation of the model and its performance. Sets of input data were created which consisted of cephalometric radiographs and a corresponding pair of coordinates (X, horizontal; Y, vertical) indicating the exact location of each landmark.

### Test dataset

A total of 460 cephalometric radiographs were used as the test dataset. The performance of the developed CNN was tested on a versatile data consisting of images with various radiographic features (such as fixed orthodontic appliances, osteosynthesis plates and others) and anatomical structures of patients at different stages of growth. The data were divided into independent subgroups to investigate the impact of the distinctive characteristics (Table 3). Radiographs of patients with mixed dentition and fixed orthodontic appliances were not included in the comparative analysis between the subgroups themselves. However, since they were part of the test data, they were included in the overall assessment of performance on the model. Similar to the training datasets, an input was created consisting of the cephalometric radiograph and a corresponding coordinate pair (X, Y) indicating the location of each landmark.

**Table 3:** Detailed Summary and distribution of the dataset used for the training and testing of the CNN

**Subgroup characteristics**			**Train Dataset**		**Test Dataset**	
No fixed orthodontic appliances	Permanent Dentition	Group I/1	175	40%	141	31%
No fixed orthodontic appliances	Mixed Dentition	Group I/2	110	26%	164	36%
Orthodontic brackets and/or bands and other fixed orthodontic appliances	Permanent Dentition	Group II	134	31%	135	29%
Orthodontic brackets and/or bands and other fixed orthodontic appliances	Mixed Dentition	Not included in the comparative analysis	11	3%	20	4%
***Total Number of images (n)***			***430***		***460***	

### Data preparation and processing

For each case, one lateral cephalogram without annotations of reference points and one with identified as well as validated cephalometric landmarks were manually exported from the database. These were anonymised, labelled as a pair, and stored in two folders. For each case, the X and Y coordinates of all marked cephalometric reference points were automatically exported from the annotated X-ray image using a custom Python script and stored in a text file (.txt) labelled to match the corresponding case.

Subsequently, the text files were automatically filtered so that only a single pair of coordinates corresponding to a specific reference point was stored in a text file. Since the location of each reference point is distinct, a Python script was written for the extraction procedure for each of the 16 mentioned landmarks. Finally, the plain cephalograms and the text document (.txt) storing a pair of coordinates were used as input for the CNN models, dealing with each point independently.

The evaluation of the accuracy of the CNN model was also performed automatically using a Python script. By this means, the trained model was accessed and applied for the detection of the specific cephalometric point. The absolute difference between the predicted point (the point identified by the CNN) and the referenced point (the point positioned by the examiner) was determined. The Results were then imported into an Excel file (Microsoft Excel for Office 365, version 16.60, Microsoft Corporation, Redmond, WA, USA) where further statistical analysis was performed.

### Model, model parameters, training and evaluation

A deep learning model, more specifically a CNN was constructed using the open-source deep learning frameworks Keras (Version 2.2.4, François Chollet) [42] and TensorFlow (Version 1.14.0, Google Brain Team) [43] accessed from a Python (Python Version 3.5.6, Python Software Foundation, Beaverton, USA) script running on NVIDIA GeForce RTX 2080 graphics card (NVIDIA, Santa Clara, CA, USA) for each of the previously mentioned landmarks (Table 2).

**Fig. 1:**
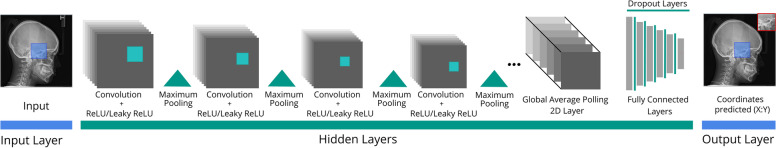
The architecture of the CNN proposed for automated cephalometric landmark recognition

The model had a commonly used CNN Architecture for image classification [39], with some custom modification. In the following, the essential components and the architecture of the framework are described as shown in Fig. 1.

The input for the training of the proposed network involved a lateral cephalogram from the training dataset and a corresponding file containing the location for the cephalometric landmarks as pairs of X and Y coordinates. Consequently, the output of the CNN was a predicted pair of X, Y coordinates indicating the position of the landmark. For example, the Training Set for the Sella Point included 430 unmarked cephalograms and 430 corresponding text files containing the position of the landmark written as a pair of X and Y coordinates. This data was processed by a convolutional layer, which detects specific features and patterns. After a feature is detected, the information is compressed and passed to the next layers of the network. This process, which is responsible for pattern recognition, is called filtering and the filters used are adjusted throughout the learning process to benefit the performance of the machine learning model. The number of filters varies for each layer, with the first layer having 30 filters, the second 60 filters continuing in ascending order for each additional layer. The learning rate was set to 10 ^−4^, batch size at 32 and as the number of layers was not consistent for each landmark. A detailed summary of the model for each point is provided in Supplementary file 1, code and data are available at Open Data LMU Platform under https://doi.org/10.5282/ubm/data.359. The performance in this model was measured with a mean squared error (MSE) cost function that quantifies the error between the real coordinates(input) of the landmark and the predicted ones(output).

In order to increase the capacity of the model, a non-linear activation function was applied after each convolutional layer (Fig. 1). To avoid the vanishing gradient problem [30] and accelerate the training speed of the neural network, a rectified linear activation function ReLU (f(x) = max(0,x)) or Leaky ReLU (f(x) = 1(x < 0)(αx) + 1(x >  = 0)(x)) where α = 0.5, depending on the outcome, was chosen. Further, a maximum pooling approach was used, which calculates the maximum value in each feature map and highlights the most frequently occurring feature in the pathway (Fig. 1).

Following the stack of convolutional layers, a global average polling 2D layer was added. This layer reduced the dimensionality of the learned feature maps by averaging over the special dimensions of the output and yielding a fixed-size vector representation, which can be processed with standard fully-connected layers.

Note, that the described composite neural architecture is comparatively large. A major problem in large neural networks is overfitting, that is, the danger of overspecialization to the training set and the resulting limited generalizability to new (upcoming) data. Regularization is a common technique to prevent overfitting and thus poor generalization performance of deep neural networks [44] To regularize the used model, dropout layers were added between the fully-connected layers of the neural network architecture (Fig. 1). Dropout is a commonly used regularization technique by which the system takes out a portion of the trainable parameters and temporarily removes them from the network, along with all incoming and outgoing connections [45].

Additionally, to optimize the performance of the model, each point was considered separately, and a modified CNN was created for each landmark. This made it possible to adapt the number of convolutional layers, filters, dropout layers, and activation functions for each landmark depending on the complexity and variety of the features, resulting in a different neural expert for each landmark. This study relied on a standard CNN architecture [39], which showed high accuracy after optimization. Therefore, an extensive hyperparameter search for neural network parameters was not conducted. However, a grid search was performed over a reasonable range of learning rates and optimizer sets without observing noticeable performance differences.

### Validation

In order to quantify the utility of the model, the absolute difference between the predicted point (the point identified by the CNN) and the referenced point (the point positioned by the examiner) was determined along the X-axis ( $\Delta {x}_{i}$) and correspondingly, along the Y-axis ($\Delta {y}_{i}$). This value was defined as Distance Error (DE) ${D}_{i}=\sqrt{ \Delta {x}_{i}^{2}+\Delta {y}_{i}^{2}}$ and it was measured across the entire dataset of test images. To be consistent with the evaluation metrics of previous studies [9, 10, 23, 37, 46], the mean radial error (MRE) along with Standard Deviation (SD) for each landmark was determined as follows:

$$MRE= \frac{{\sum }_{i=1}^{n}Di}{n}$$

and $SD= \sqrt{\frac{{\sum }_{i=1}^{n}{\left(Di-\sqrt{ \Delta {x}^{2}- \Delta {y}^{2}}\right)}^{2}}{n-1}}$ where $n$ is the total amount of images.

The Successful Detection Rate (SDR), which indicates the percentage of correctly detected reference points in different precision ranges: $$SD{R}_{z}= \frac{number of accurate detection}{number of detection} \times 100\%$$ was computed, specifying four types of accuracy ranges: $$z$$ = 2.0 mm, 2.5 mm, 3.0 mm, 4.0 mm.

It should be considered that the deviation of the distance error along a certain axis has greater importance for some points. For example, the accuracy of the B-Point along the X-axis is more significant as it marks the position of the mandible in the sagittal plane. Hence, the distribution of errors in the horizontal and vertical planes were considered separately.

### Statistical analysis

Mean radial errors and standard deviations of the 16 used orthodontic cephalometric reference points were collected in an excel file (Microsoft Excel for Office 365, Version 16.60, Microsoft Corporation, Redmond, WA, USA). These numbers were categorized in two main comparing groups:

I No fixed orthodontic appliances—Permanent dentition XY – Error (Group I/1) versus No Fixed orthodontic appliances—Mixed dentition XY – Error (Group I/2)
II No fixed orthodontic appliances—Permanent dentition XY – Error (Group I/1) versus Orthodontic brackets and/or bands and other fixed orthodontic appliances—Permanent dentition XY – Error (Group II)

A t-test was applied to compare MREs with their corresponding SDs for each landmark in their two categories, to determine whether the means of these two groups are equal to each other. For this purpose, a t-test was run for both abovementioned categories (Table 4), separately for all the 16 points (an overall of 32 tests). All data were analyzed using R software (Version R-4.1.1, R Development Core Team, Vienna, Austria). Statistical significance was set at a *p*-value < 0.05.

**Table 4:** Equation applied for statistical evaluation

$t=\frac{\overline{{x }_{1}}-\overline{{x }_{2}}}{\sqrt{\frac{{s}_{1}^{2}}{{n}_{1}}+\frac{{s}_{2}^{2}}{{n}_{2}}}}$	t: t-value
	$\overline{{x }_{1}}$: Mean value of the first group
	$\overline{{x }_{2}}$: Mean value of the second group
	${n}_{1}:$ Size of the first group
	${n}_{2}$: Size of the second group
	${s}_{1}$: Standard deviation of the first group
	${s}_{2}$: Standard deviation of the second group

## Results

The results for the different groups are presented in Table 5. Statistically significant differences were observed in the recognition accuracy of the Ap-Inferior point and the Is-Superior point between patients with permanent dentition (I/1) and mixed dentition (I/2), both without fixed orthodontic appliances. No statistically significant differences were found in the recognition process between patients without fixed orthodontic appliances (I/1) and patients with orthodontic brackets and/or bands and/or other fixed orthodontic appliances (II), both examined in the permanent dentition only. The overall performance of the model showed higher MRE and SD in group I/2, suggesting lower accuracy in such conditions. The highest accuracy was obtained in group II, however without statistically significant differences from group I/1.

**Table 5:** Comparison of model performance across different patient groups

	**I. No fixed orthodontic appliances**					**II. Orthodontic brackets and/or bands and other fixed orthodontic appliances**		
	**I/1** **Permanent Dentition**		**I/2** **Mixed Dentition**		**T-Test** **Between Groups I/1 and I/2**	**II** **Permanent Dentition**		**T-Test** **Between Groups I/1 and II**
**N**	**141**		**164**			**135**		
	**MRE (mm)**	**SD (mm)**	**MRE (mm)**	**SD (mm)**	***p value***	**MRE (mm)**	**SD (mm)**	***p value***
A-Point	1.21	0.71	1.31	0.76	0.25	1.31	0.84	0.30
Ap-Superior	1.90	1.21	2.15	1.52	0.12	1.72	1.07	0.19
Ap-Inferior	1.72	1.12	2.32	1.63	** < 0.01**	1.61	0.97	0.35
ANS	1.72	1.14	1.77	1.76	0.78	1.66	1.31	0.66
Art	1.24	1.00	1.25	0.72	0.93	1.20	0.79	0.72
B-Point	1.24	0.60	1.25	0.70	0.90	1.27	0.74	0.77
Ba	1.66	1.15	1.69	1.19	0.83	1.53	1.01	0.32
Me	1.37	0.89	1.46	0.92	0.40	1.25	0.95	0.28
Is-Superior	0.86	0.50	1.20	1.15	** < 0.01**	0.88	0.44	0.64
Is-Inferior	1.14	0.71	1.24	1.22	0.39	1.18	0.73	0.64
N	1.16	0.67	1.32	0.92	0.07	1.12	0.64	0.60
Pog	1.63	1.13	1.80	1.44	0.25	1.52	0.96	0.35
PNS	1.68	1.27	1.71	1.45	0.87	1.59	1.10	0.54
S	1.01	0.64	0.93	0.63	0.31	0.89	0.53	0.10
T1	1.71	1.29	1.60	1.20	0.45	1.54	1.14	0.27
T2	1.68	1.09	1.75	1.23	0.57	1.64	1.12	0.77
**AVERAGE**	**1.43**	**0.95**	**1.55**	**1.15**		**1.37**	**0.90**	

The descriptive statistics indicating the mean error in the X-axis and Y-axis, the mean radial error and standard deviation, and the SDR (accuracy ranges: z = 2.0 mm, 2.5 mm, 3.0 mm, 4.0 mm) for all studied groups of patients are shown in Table 6. The proposed model exhibited an overall mean radial error (MRE) of 1.47 mm with a standard deviation of 1.06 mm. The results revealed a successful detection rate (SDR) of 84.73%, 88.21%, 91.94%, and 96.58% in the range of 2, 2.5, 3, and 4 mm, respectively. The Sella point demonstrated the lowest MRE and highest SDR values, whereas the Ap-Superior point had the highest MRE and lowest SDR values. The PNS point showed the smallest mean error on the Y-axis but the largest on the X-axis.

**Table 6:** Overall model performance for all observed patient groups

	**Mean** **X -Error**	**Mean** **Y -Error**	**Mean Radial Error**	**Standard Deviation**	**SDR (Successful detection rates) %**			
	**(mm)**	**(mm)**	**MRE** **(mm)**	**SD** **(mm)**	**SDR %** ***2 mm***	**SDR %** ***2.5 mm***	**SDR %** ***3 mm***	**SDR %** ***4 mm***
A-Point	0.74	0.90	1.29	0.77	89.57	93.91	96.09	99.13
Ap-Superior	1.08	1.38	1.93	1.30	66.74	75.43	84.57	93.48
Ap-Inferior	1.07	1.40	1.91	1.34	67.17	75.00	83.48	93.70
ANS	1.21	0.96	1.75	1.51	80.65	81.96	87.39	94.57
Art	0.79	0.79	1.25	0.85	89.57	92.39	95.87	98.48
B-Point	0.55	1.04	1.26	0.81	94.13	95.65	96.52	99.35
Ba	0.90	1.18	1.66	1.21	86.09	86.74	90.43	94.78
Me	1.00	0.75	1.38	0.93	85.87	89.57	93.91	97.39
Is-Superior	0.59	0.68	1.00	0.80	93.91	96.96	97.83	98.91
Is-Inferior	0.67	0.84	1.19	0.93	89.57	94.57	96.30	99.35
N	0.67	0.86	1.21	0.77	88.70	94.57	98.04	99.57
Pog	0.65	1.39	1.66	1.22	79.35	82.17	88.48	95.00
PNS	1.31	0.83	1.71	1.34	82.39	83.48	85.65	93.04
S	0.63	0.59	0.97	0.84	94.13	97.17	98.04	99.57
T1	1.00	1.02	1.61	1.21	83.26	85.00	89.35	94.57
T2	1.28	0.91	1.72	1.18	84.57	86.74	89.13	94.35
**AVERAGE**			**1.47**	**1.06**	**84.73**	**88.21**	**91.94**	**96.58**

## Discussion

The null hypothesis that developmental stages of a dentition, fixed orthodontic appliances or other dental appliances do not affect the accuracy of a customized artificial model for automatic detection of cephalometric landmarks was partially confirmed. The results of this study indicated that fixed orthodontic appliances had no significant impact on the recognition of cephalometric landmarks. However, growth structures such as tooth germs in the mixed dentition affected the performance of the studied model.

Images of patients with permanent dentition showed showed homogeneous anatomical patterns in the areas of the landmarks to be placed. In contrast, patients with mixed dentition were associated with complex growth structures, varying bone density and uniquely positioned permanent tooth germs. Consequently, the recognition process showed better accuracy for images of patients with permanent dentition, while the overall performance of the model was lower for cases with mixed dentition (Table 5). As the most common sequence of eruption is the lower central incisor, followed by the permanent molars, the upper central incisors and the lower lateral incisor, the radiographic appearance of the cephalometric landmarks marking the dental structures in this area may vary greatly depending on the stage of development of the permanent teeth as well as the extent of resorption of the roots of the deciduous teeth. In addition, a temporary stage of crowding of the incisors can be expected in the early mixed dentition [47], which may lead to the appearance of double contours, superimpositions, and density differences between adjacent regions. In this study, the impact of growth structures on the recognition process of the cephalometric landmarks marking dental structures in mixed dentition patients was found to be statistically significant (*p* < 0.05) observed in the MREs of Ap-Inferior Point and Is-Superior Point. A recent study also reported a lower accuracy rate of the detection of the root apices [25], however the tips of the incisal edges of the incisors were not associated with any recognition difficulties. The possible reason for this difference could be related to the stages of a mixed dentition. However, the data sample of adolescent patients in this study was categorized as mixed dentition, which included both early mixed dentition and late mixed dentition.

In order to eliminate the complexity of growth structures, the influence of fixed orthodontic appliances on the model’s performance was studied only in patients with permanent dentition. Cephalometric radiographs with fixed orthodontic appliances are usually obtained at a later stage of treatment when initial objectives such as crowding, eruption problems, impacted teeth, and occlusal relationship problems have been resolved. Since at this stage of orthodontic treatment the teeth are usually well aligned, the overall detection of cephalometric points is less affected by double contours and superimpositions, but may be affected by metal artifacts. In this study, the overall detection of cephalometric points was more accurate for images of patients with fixed orthodontic appliances, and there was no significant difference in accuracy between cephalometric radiographs of patients with orthodontic brackets and/or bands and other fixed orthodontic appliances and cephalometric radiographs of patients without fixed orthodontic appliances. It should be noted that common fixed orthodontic appliances are made of stainless steel or other alloys and therefore have a different radiographic density than skeletal structures. A similar pattern of results may be seen in of radiographs with other factors associated with comparable density that may affect the performance of the framework, such as artifacts, osteosynthesis plates, implants, prosthetic restorations, and root canal fillings. Nevertheless, the present study did not investigate these aspects due to the limited study data.

The distribution of errors in the horizontal and vertical planes was considered independently of each other. By means of a common cephalometric appraisal, the anteroposterior or vertical position of the maxilla and mandible and their relationships to the cranial base and dental structures are evaluated. For this purpose, the image was considered as a coordinate system with its two axes: X and Y. Transferred to the lateral cephalogram, these mark the sagittal and vertical planes respectively. The results for cephalometric points marking important anteroposterior correlations, such as A point and B point, showed overall a smaller distance error on the X axis than on the Y axis (Table 6). It is at the reference points marking the positions of the skeletal structures in the sagittal plane that an error on the X-axis would be of greater clinical significance, as has been noted in a recent scoping review [37]. Equivalently, the results for cephalometric points, such as PNS and ANS, marking larger vertical correlations showed comparable results in terms of distance error on the Y-axis, which is more clinically relevant in this case.

Differences in landmark recognition in the X-axis or Y-axis can be explained by the fact that each landmark is located at a distinct anatomical site that is more accurate to locate in either the vertical or horizontal direction [4]. Especially bilateral landmarks might show higher deviations in the Y-axis due to double contours associated with motion artifacts or incorrect positioning [1]. Lastly, the annotation method used in the present study may be prone to error due to interrater and intrarater variability and may also have contributed to differences in recognition in one direction or the other.

Investigating the distinct characteristics and the exact position of the dentoskeletal landmarks is essential for the quality of the cephalometric appraisal. Therefore, the focus was set on developing an independent CNN type suitable for the unique characteristics of each reference point. This approach eliminated the expected decline in accuracy with increasing number of detection targets described in a previous study [48]. The number of convolutional layers, filters, dropout layers and activation functions were adjusted for each landmark depending on its anatomical complexity. The results of the present study in terms of MRE (1.47) and SD (1.06 mm) are generally consistent with those of previous studies [10, 14, 16, 18, 22, 49]. However, as both training and test data differ, an objective comparison is not possible. One limitation of the proposed CNN architecture is that it lacks uncertainty quantification [50]. Future research may distinguish between aleatoric (irreducible) and epistemic (reducible) uncertainty. The latter can be especially beneficial in the small to moderate data regime. Having established the feasibility of the method in cephalometric landmark detection of patients with fixed appliances and the underlying challenges in patients with mixed dentition, future research could focus on systematically comparing the performance of more advanced models, such as those based on ResNet or DenseNet [51–53] and improving network architectures (e.g., by applying Bayesian optimization techniques, [54]).

Although the performance of the developed CNN was tested on a versatile dataset consisting of images with a variety of radiological features, this study employed a relatively small dataset for images, particularly from Group III (Others (artifacts, osteosynthesis plates, implants, dental prosthetic restorations and root canal treatments)). Indeed, the challenge of limited training data in the health sector was also recognized in a recent review on deep learning [39]. Hence, following research based on larger and well-balanced datasets is needed to assess the specifics of these parameters.

Another limitation of this study is the annotation procedure used, as it is prone to error with regard to the examiner. In the absence of a gold standard, constructing a reliable reference test capable of reducing bias in the dataset remains a challenge [40].

Finally, since the reference points are used in a further step of the cephalometric analysis to perform angular measurements [52], a potential limitation of the proposed framework is that such measurements and index data were not obtained. Nevertheless, it should be noted that the cephalometric angles depend to a large extent on the correct positioning of the reference points. Future studies should address the aspect of the angular measurements to assess the suitability of automated cephalometric landmark recognition for clinical use.

## Conclusions

The radiographic appearance of fixed orthodontic appliances such as brackets, bands, and other fixed orthodontic appliances on a lateral cephalometric radiograph did not significantly influence the performance of the model. Complex growth structures may affect the recognition accuracy of dental landmarks, thus detected references should be verified in growing patients and in the mixed dentition.

## Supplementary Information

**Additional file 1.**

## Data Availability

Data and material are available on Open Data LMU Platform under the following https://doi.org/10.5282/ubm/data.359.

## Abbreviations

CNN: Convolutional neural networks
MRE: Mean radial error
SDR: Successful detection rate
NNs: Neural networks
MSE: Mean squared error
SD: Standard Deviation
SDR: Successful Detection Rate
