# Supplementary material for: Influence of growth structures and fixed appliances on automated cephalometric landmark recognition with a customized convolutional neural network
Source: BMC Oral Health. 2023 May 10;23:274. doi: 10.1186/s12903-023-02984-2 (PMC10173502; doi:10.1186/s12903-023-02984-2)
Supplement: Supplementary file 1 — Additional file 1. [file 12903_2023_2984_MOESM1_ESM.pdf]

Detailed model summary for each CNN applied for the referenced cephalometric landmarks.

batch size = 32
learning rate = 10^{-4}

Models summary

A-Point - Subspinale

Layer (type)	Output Shape	Param #
conv2d_1 (Conv2D)	(None, 517, 517, 30)	510
max_pooling2d_1 (MaxPooling2	(None, 258, 258, 30)	0
conv2d_2 (Conv2D)	(None, 255, 255, 60)	28860
conv2d_3 (Conv2D)	(None, 252, 252, 90)	86490
max_pooling2d_2 (MaxPooling2	(None, 126, 126, 90)	0
conv2d_4 (Conv2D)	(None, 123, 123, 120)	172920
conv2d_5 (Conv2D)	(None, 120, 120, 150)	288150
max_pooling2d_3 (MaxPooling2	(None, 60, 60, 150)	0
conv2d_6 (Conv2D)	(None, 57, 57, 180)	432180
conv2d_7 (Conv2D)	(None, 54, 54, 210)	605010
batch_normalization_1 (Batch	(None, 54, 54, 210)	840
max_pooling2d_4 (MaxPooling2	(None, 27, 27, 210)	0
conv2d_8 (Conv2D)	(None, 24, 24, 240)	806640
conv2d_9 (Conv2D)	(None, 21, 21, 240)	921840
max_pooling2d_5 (MaxPooling2	(None, 10, 10, 240)	0
conv2d_10 (Conv2D)	(None, 7, 7, 480)	1843680
conv2d_11 (Conv2D)	(None, 4, 4, 480)	3686880

```
_________________________________________________________________
global_average_pooling2d_1 ( (None, 480)              0
_________________________________________________________________
dense_1 (Dense)              (None, 600)              288600
_________________________________________________________________
leaky_re_lu_1 (LeakyReLU)    (None, 600)              0
_________________________________________________________________
dropout_1 (Dropout)          (None, 600)              0
_________________________________________________________________
dense_2 (Dense)              (None, 400)              240400
_________________________________________________________________
dropout_2 (Dropout)          (None, 400)              0
_________________________________________________________________
dense_3 (Dense)              (None, 500)              200500
_________________________________________________________________
dropout_3 (Dropout)          (None, 500)              0
_________________________________________________________________
dense_4 (Dense)              (None, 400)              200400
_________________________________________________________________
dropout_4 (Dropout)          (None, 400)              0
_________________________________________________________________
dense_5 (Dense)              (None, 300)              120300
_________________________________________________________________
dropout_5 (Dropout)          (None, 300)              0
_________________________________________________________________
dense_6 (Dense)              (None, 200)              60200
_________________________________________________________________
dropout_6 (Dropout)          (None, 200)              0
_________________________________________________________________
dense_7 (Dense)              (None, 100)              20100
_________________________________________________________________
dense_8 (Dense)              (None, 50)               5050
_________________________________________________________________
dense_9 (Dense)              (None, 2)                102
=================================================================
Total params: 10,009,652
Trainable params: 10,009,232
Non-trainable params: 420
```

Ap 1 - Apex superior

```
_________________________________________________________________
Layer (type)                 Output Shape              Param #
=================================================================
conv2d_1 (Conv2D)            (None, 517, 517, 30)      510
```

max_pooling2d_1 (MaxPooling2	(None, 258, 258, 30)	0
conv2d_2 (Conv2D)	(None, 255, 255, 60)	28860
conv2d_3 (Conv2D)	(None, 252, 252, 90)	86490
max_pooling2d_2 (MaxPooling2	(None, 126, 126, 90)	0
conv2d_4 (Conv2D)	(None, 123, 123, 120)	172920
conv2d_5 (Conv2D)	(None, 120, 120, 150)	288150
max_pooling2d_3 (MaxPooling2	(None, 60, 60, 150)	0
conv2d_6 (Conv2D)	(None, 57, 57, 180)	432180
conv2d_7 (Conv2D)	(None, 54, 54, 210)	605010
batch_normalization_1 (Batch	(None, 54, 54, 210)	840
max_pooling2d_4 (MaxPooling2	(None, 27, 27, 210)	0
conv2d_8 (Conv2D)	(None, 24, 24, 240)	806640
conv2d_9 (Conv2D)	(None, 21, 21, 240)	921840
max_pooling2d_5 (MaxPooling2	(None, 10, 10, 240)	0
conv2d_10 (Conv2D)	(None, 7, 7, 480)	1843680
conv2d_11 (Conv2D)	(None, 4, 4, 480)	3686880
global_average_pooling2d_1 (	(None, 480)	0
dense_1 (Dense)	(None, 600)	288600
leaky_re_lu_1 (LeakyReLU)	(None, 600)	0
dropout_1 (Dropout)	(None, 600)	0
dense_2 (Dense)	(None, 400)	240400
leaky_re_lu_2 (LeakyReLU)	(None, 400)	0

```
_________________________________________________________________
dropout_2 (Dropout)          (None, 400)               0
_________________________________________________________________
leaky_re_lu_3 (LeakyReLU)    (None, 400)               0
_________________________________________________________________
dropout_3 (Dropout)          (None, 400)               0
_________________________________________________________________
dense_3 (Dense)              (None, 400)               160400
_________________________________________________________________
dropout_4 (Dropout)          (None, 400)               0
_________________________________________________________________
dense_4 (Dense)              (None, 300)               120300
_________________________________________________________________
dropout_5 (Dropout)          (None, 300)               0
_________________________________________________________________
dense_5 (Dense)              (None, 200)               60200
_________________________________________________________________
dropout_6 (Dropout)          (None, 200)               0
_________________________________________________________________
dense_6 (Dense)              (None, 100)               20100
_________________________________________________________________
dense_7 (Dense)              (None, 50)                5050
_________________________________________________________________
dense_8 (Dense)              (None, 2)                 102
=================================================================
Total params: 9,769,152
Trainable params: 9,768,732
Non-trainable params: 420
```

Ap $\bar{1}$ - Apex inferior

```
_________________________________________________________________
Layer (type)                 Output Shape              Param #
=================================================================
conv2d_1 (Conv2D)            (None, 517, 517, 30)      510
_________________________________________________________________
max_pooling2d_1 (MaxPooling2 (None, 258, 258, 30)      0
_________________________________________________________________
conv2d_2 (Conv2D)            (None, 255, 255, 60)      28860
_________________________________________________________________
conv2d_3 (Conv2D)            (None, 252, 252, 90)      86490
_________________________________________________________________
max_pooling2d_2 (MaxPooling2 (None, 126, 126, 90)      0
_________________________________________________________________
conv2d_4 (Conv2D)            (None, 123, 123, 120)     172920
```

conv2d_5 (Conv2D)	(None, 120, 120, 150)	288150
max_pooling2d_3 (MaxPooling2	(None, 60, 60, 150)	0
conv2d_6 (Conv2D)	(None, 57, 57, 180)	432180
conv2d_7 (Conv2D)	(None, 54, 54, 210)	605010
batch_normalization_1 (Batch	(None, 54, 54, 210)	840
max_pooling2d_4 (MaxPooling2	(None, 27, 27, 210)	0
conv2d_8 (Conv2D)	(None, 24, 24, 240)	806640
conv2d_9 (Conv2D)	(None, 21, 21, 240)	921840
max_pooling2d_5 (MaxPooling2	(None, 10, 10, 240)	0
conv2d_10 (Conv2D)	(None, 7, 7, 480)	1843680
conv2d_11 (Conv2D)	(None, 4, 4, 480)	3686880
global_average_pooling2d_1 (	(None, 480)	0
dense_1 (Dense)	(None, 600)	288600
dropout_1 (Dropout)	(None, 600)	0
dense_2 (Dense)	(None, 400)	240400
dropout_2 (Dropout)	(None, 400)	0
dense_3 (Dense)	(None, 500)	200500
dropout_3 (Dropout)	(None, 500)	0
dense_4 (Dense)	(None, 400)	200400
dropout_4 (Dropout)	(None, 400)	0
dense_5 (Dense)	(None, 300)	120300
dropout_5 (Dropout)	(None, 300)	0

```
_________________________________________________________________
dense_6 (Dense)              (None, 200)               60200
_________________________________________________________________
dropout_6 (Dropout)          (None, 200)               0
_________________________________________________________________
dense_7 (Dense)              (None, 100)               20100
_________________________________________________________________
dense_8 (Dense)              (None, 50)                5050
_________________________________________________________________
dense_9 (Dense)              (None, 2)                 102
=================================================================
Total params: 10,009,652
Trainable params: 10,009,232
Non-trainable params: 420
```

ANS - Anterior nasal spine

```
_________________________________________________________________
Layer (type)                 Output Shape              Param #
=================================================================
conv2d_1 (Conv2D)            (None, 517, 517, 30)      510
_________________________________________________________________
max_pooling2d_1 (MaxPooling2 (None, 258, 258, 30)      0
_________________________________________________________________
conv2d_2 (Conv2D)            (None, 255, 255, 60)      28860
_________________________________________________________________
conv2d_3 (Conv2D)            (None, 252, 252, 90)      86490
_________________________________________________________________
max_pooling2d_2 (MaxPooling2 (None, 126, 126, 90)      0
_________________________________________________________________
conv2d_4 (Conv2D)            (None, 123, 123, 120)     172920
_________________________________________________________________
conv2d_5 (Conv2D)            (None, 120, 120, 150)     288150
_________________________________________________________________
max_pooling2d_3 (MaxPooling2 (None, 60, 60, 150)       0
_________________________________________________________________
conv2d_6 (Conv2D)            (None, 57, 57, 180)       432180
_________________________________________________________________
conv2d_7 (Conv2D)            (None, 54, 54, 210)       605010
_________________________________________________________________
batch_normalization_1 (Batch (None, 54, 54, 210)       840
_________________________________________________________________
max_pooling2d_4 (MaxPooling2 (None, 27, 27, 210)       0
_________________________________________________________________
conv2d_8 (Conv2D)            (None, 24, 24, 240)       806640
```

conv2d_9 (Conv2D)	(None, 21, 21, 240)	921840
max_pooling2d_5 (MaxPooling2	(None, 10, 10, 240)	0
conv2d_10 (Conv2D)	(None, 7, 7, 480)	1843680
conv2d_11 (Conv2D)	(None, 4, 4, 480)	3686880
global_average_pooling2d_1 (	(None, 480)	0
dense_1 (Dense)	(None, 600)	288600
leaky_re_lu_1 (LeakyReLU)	(None, 600)	0
dropout_1 (Dropout)	(None, 600)	0
dense_2 (Dense)	(None, 400)	240400
leaky_re_lu_2 (LeakyReLU)	(None, 400)	0
dropout_2 (Dropout)	(None, 400)	0
leaky_re_lu_3 (LeakyReLU)	(None, 400)	0
dropout_3 (Dropout)	(None, 400)	0
dense_3 (Dense)	(None, 400)	160400
dropout_4 (Dropout)	(None, 400)	0
dense_4 (Dense)	(None, 300)	120300
dropout_5 (Dropout)	(None, 300)	0
dense_5 (Dense)	(None, 200)	60200
dropout_6 (Dropout)	(None, 200)	0
dense_6 (Dense)	(None, 100)	20100
dense_7 (Dense)	(None, 50)	5050
dense_8 (Dense)	(None, 2)	102

===
Total params: 9,769,152
Trainable params: 9,768,732
Non-trainable params: 420

Art – Articulare

____

Layer (type)	Output Shape	Param #
conv2d_1 (Conv2D)	(None, 517, 517, 30)	510
max_pooling2d_1 (MaxPooling2	(None, 258, 258, 30)	0
conv2d_2 (Conv2D)	(None, 255, 255, 60)	28860
conv2d_3 (Conv2D)	(None, 252, 252, 90)	86490
max_pooling2d_2 (MaxPooling2	(None, 126, 126, 90)	0
conv2d_4 (Conv2D)	(None, 123, 123, 120)	172920
conv2d_5 (Conv2D)	(None, 120, 120, 150)	288150
max_pooling2d_3 (MaxPooling2	(None, 60, 60, 150)	0
conv2d_6 (Conv2D)	(None, 57, 57, 180)	432180
conv2d_7 (Conv2D)	(None, 54, 54, 210)	605010
batch_normalization_1 (Batch	(None, 54, 54, 210)	840
max_pooling2d_4 (MaxPooling2	(None, 27, 27, 210)	0
conv2d_8 (Conv2D)	(None, 24, 24, 240)	806640
conv2d_9 (Conv2D)	(None, 21, 21, 240)	921840
max_pooling2d_5 (MaxPooling2	(None, 10, 10, 240)	0
conv2d_10 (Conv2D)	(None, 7, 7, 480)	1843680
conv2d_11 (Conv2D)	(None, 4, 4, 480)	3686880
global_average_pooling2d_1 (	(None, 480)	0

```
_________________________________________________________________
dense_1 (Dense)            (None, 600)            288600
_________________________________________________________________
leaky_re_lu_1 (LeakyReLU)    (None, 600)            0
_________________________________________________________________
dropout_1 (Dropout)         (None, 600)            0
_________________________________________________________________
dense_2 (Dense)            (None, 400)            240400
_________________________________________________________________
dropout_2 (Dropout)         (None, 400)            0
_________________________________________________________________
dense_3 (Dense)            (None, 500)            200500
_________________________________________________________________
dropout_3 (Dropout)         (None, 500)            0
_________________________________________________________________
dense_4 (Dense)            (None, 400)            200400
_________________________________________________________________
dropout_4 (Dropout)         (None, 400)            0
_________________________________________________________________
dense_5 (Dense)            (None, 300)            120300
_________________________________________________________________
dropout_5 (Dropout)         (None, 300)            0
_________________________________________________________________
dense_6 (Dense)            (None, 200)            60200
_________________________________________________________________
dropout_6 (Dropout)         (None, 200)            0
_________________________________________________________________
dense_7 (Dense)            (None, 100)            20100
_________________________________________________________________
dense_8 (Dense)            (None, 50)             5050
_________________________________________________________________
dense_9 (Dense)            (None, 2)              102
=================================================================
Total params: 10,009,652
Trainable params: 10,009,232
Non-trainable params: 420
```

B-Point – Supramentale

```
_________________________________________________________________
Layer (type)              Output Shape            Param #
=================================================================
conv2d_1 (Conv2D)          (None, 517, 517, 30)      510
_________________________________________________________________
max_pooling2d_1 (MaxPooling2 (None, 258, 258, 30)      0
```

conv2d_2 (Conv2D)	(None, 255, 255, 60)	28860
conv2d_3 (Conv2D)	(None, 252, 252, 90)	86490
max_pooling2d_2 (MaxPooling2	(None, 126, 126, 90)	0
conv2d_4 (Conv2D)	(None, 123, 123, 120)	172920
conv2d_5 (Conv2D)	(None, 120, 120, 150)	288150
max_pooling2d_3 (MaxPooling2	(None, 60, 60, 150)	0
conv2d_6 (Conv2D)	(None, 57, 57, 180)	432180
conv2d_7 (Conv2D)	(None, 54, 54, 210)	605010
batch_normalization_1 (Batch	(None, 54, 54, 210)	840
max_pooling2d_4 (MaxPooling2	(None, 27, 27, 210)	0
conv2d_8 (Conv2D)	(None, 24, 24, 240)	806640
conv2d_9 (Conv2D)	(None, 21, 21, 240)	921840
max_pooling2d_5 (MaxPooling2	(None, 10, 10, 240)	0
conv2d_10 (Conv2D)	(None, 7, 7, 480)	1843680
conv2d_11 (Conv2D)	(None, 4, 4, 480)	3686880
global_average_pooling2d_1 (	(None, 480)	0
dense_1 (Dense)	(None, 600)	288600
dropout_1 (Dropout)	(None, 600)	0
dense_2 (Dense)	(None, 400)	240400
dropout_2 (Dropout)	(None, 400)	0
dense_3 (Dense)	(None, 500)	200500
dropout_3 (Dropout)	(None, 500)	0

```
_________________________________________________________________
dense_4 (Dense)              (None, 400)              200400
_________________________________________________________________
dropout_4 (Dropout)          (None, 400)              0
_________________________________________________________________
dense_5 (Dense)              (None, 300)              120300
_________________________________________________________________
dropout_5 (Dropout)          (None, 300)              0
_________________________________________________________________
dense_6 (Dense)              (None, 200)              60200
_________________________________________________________________
dropout_6 (Dropout)          (None, 200)              0
_________________________________________________________________
dense_7 (Dense)              (None, 100)              20100
_________________________________________________________________
dense_8 (Dense)              (None, 50)               5050
_________________________________________________________________
dense_9 (Dense)              (None, 2)                102
=================================================================
Total params: 10,009,652
Trainable params: 10,009,232
Non-trainable params: 420
```

Ba – Basion

```
_________________________________________________________________
Layer (type)                 Output Shape              Param #
=================================================================
conv2d_1 (Conv2D)            (None, 517, 517, 30)      510
_________________________________________________________________
max_pooling2d_1 (MaxPooling2 (None, 258, 258, 30)      0
_________________________________________________________________
conv2d_2 (Conv2D)            (None, 255, 255, 60)      28860
_________________________________________________________________
conv2d_3 (Conv2D)            (None, 252, 252, 90)      86490
_________________________________________________________________
max_pooling2d_2 (MaxPooling2 (None, 126, 126, 90)      0
_________________________________________________________________
conv2d_4 (Conv2D)            (None, 123, 123, 120)     172920
_________________________________________________________________
conv2d_5 (Conv2D)            (None, 120, 120, 150)     288150
_________________________________________________________________
max_pooling2d_3 (MaxPooling2 (None, 60, 60, 150)       0
_________________________________________________________________
conv2d_6 (Conv2D)            (None, 57, 57, 180)       432180
```

conv2d_7 (Conv2D)	(None, 54, 54, 210)	605010
batch_normalization_1 (Batch	(None, 54, 54, 210)	840
max_pooling2d_4 (MaxPooling2	(None, 27, 27, 210)	0
conv2d_8 (Conv2D)	(None, 24, 24, 240)	806640
conv2d_9 (Conv2D)	(None, 21, 21, 240)	921840
max_pooling2d_5 (MaxPooling2	(None, 10, 10, 240)	0
conv2d_10 (Conv2D)	(None, 7, 7, 480)	1843680
conv2d_11 (Conv2D)	(None, 4, 4, 480)	3686880
global_average_pooling2d_1 (	(None, 480)	0
dense_1 (Dense)	(None, 600)	288600
dropout_1 (Dropout)	(None, 600)	0
dense_2 (Dense)	(None, 500)	300500
dropout_2 (Dropout)	(None, 500)	0
dense_3 (Dense)	(None, 500)	250500
dropout_3 (Dropout)	(None, 500)	0
dense_4 (Dense)	(None, 400)	200400
dropout_4 (Dropout)	(None, 400)	0
dense_5 (Dense)	(None, 300)	120300
dropout_5 (Dropout)	(None, 300)	0
dense_6 (Dense)	(None, 200)	60200
dropout_6 (Dropout)	(None, 200)	0
dense_7 (Dense)	(None, 100)	20100

```
_________________________________________________________________
dense_8 (Dense)          (None, 50)             5050
_________________________________________________________________
dense_9 (Dense)          (None, 2)              102
=================================================================
Total params: 10,119,752
Trainable params: 10,119,332
Non-trainable params: 420
```

Me – Menton

```
_________________________________________________________________
Layer (type)             Output Shape           Param #
=================================================================
conv2d_1 (Conv2D)          (None, 517, 517, 30)      510
_________________________________________________________________
max_pooling2d_1 (MaxPooling2 (None, 258, 258, 30)      0
_________________________________________________________________
conv2d_2 (Conv2D)          (None, 255, 255, 60)      28860
_________________________________________________________________
conv2d_3 (Conv2D)          (None, 252, 252, 90)      86490
_________________________________________________________________
max_pooling2d_2 (MaxPooling2 (None, 126, 126, 90)      0
_________________________________________________________________
conv2d_4 (Conv2D)          (None, 123, 123, 120)     172920
_________________________________________________________________
conv2d_5 (Conv2D)          (None, 120, 120, 150)     288150
_________________________________________________________________
max_pooling2d_3 (MaxPooling2 (None, 60, 60, 150)       0
_________________________________________________________________
conv2d_6 (Conv2D)          (None, 57, 57, 180)       432180
_________________________________________________________________
conv2d_7 (Conv2D)          (None, 54, 54, 210)       605010
_________________________________________________________________
batch_normalization_1 (Batch (None, 54, 54, 210)       840
_________________________________________________________________
max_pooling2d_4 (MaxPooling2 (None, 27, 27, 210)       0
_________________________________________________________________
conv2d_8 (Conv2D)          (None, 24, 24, 240)       806640
_________________________________________________________________
conv2d_9 (Conv2D)          (None, 21, 21, 240)       921840
_________________________________________________________________
max_pooling2d_5 (MaxPooling2 (None, 10, 10, 240)       0
_________________________________________________________________
conv2d_10 (Conv2D)         (None, 7, 7, 480)        1843680
```

conv2d_11 (Conv2D)	(None, 4, 4, 480)	3686880
global_average_pooling2d_1 (	(None, 480)	0
dense_1 (Dense)	(None, 600)	288600
leaky_re_lu_1 (LeakyReLU)	(None, 600)	0
dropout_1 (Dropout)	(None, 600)	0
dense_2 (Dense)	(None, 400)	240400
dropout_2 (Dropout)	(None, 400)	0
dense_3 (Dense)	(None, 500)	200500
dropout_3 (Dropout)	(None, 500)	0
dense_4 (Dense)	(None, 400)	200400
dropout_4 (Dropout)	(None, 400)	0
dense_5 (Dense)	(None, 300)	120300
dropout_5 (Dropout)	(None, 300)	0
dense_6 (Dense)	(None, 200)	60200
dropout_6 (Dropout)	(None, 200)	0
dense_7 (Dense)	(None, 100)	20100
dense_8 (Dense)	(None, 50)	5050
dense_9 (Dense)	(None, 2)	102

===

Total params: 10,009,652
Trainable params: 10,009,232
Non-trainable params: 420

Is 1 - Incision Superior

Layer (type)	Output Shape	Param #

```
=================================================================
conv2d_1 (Conv2D)           (None, 517, 517, 30)      510
_________________________________________________________________
max_pooling2d_1 (MaxPooling2 (None, 258, 258, 30)      0
_________________________________________________________________
conv2d_2 (Conv2D)           (None, 255, 255, 60)      28860
_________________________________________________________________
conv2d_3 (Conv2D)           (None, 252, 252, 90)      86490
_________________________________________________________________
max_pooling2d_2 (MaxPooling2 (None, 126, 126, 90)      0
_________________________________________________________________
conv2d_4 (Conv2D)           (None, 123, 123, 120)     172920
_________________________________________________________________
conv2d_5 (Conv2D)           (None, 120, 120, 150)     288150
_________________________________________________________________
max_pooling2d_3 (MaxPooling2 (None, 60, 60, 150)       0
_________________________________________________________________
conv2d_6 (Conv2D)           (None, 57, 57, 180)       432180
_________________________________________________________________
conv2d_7 (Conv2D)           (None, 54, 54, 210)       605010
_________________________________________________________________
batch_normalization_1 (Batch (None, 54, 54, 210)       840
_________________________________________________________________
max_pooling2d_4 (MaxPooling2 (None, 27, 27, 210)       0
_________________________________________________________________
conv2d_8 (Conv2D)           (None, 24, 24, 240)       806640
_________________________________________________________________
conv2d_9 (Conv2D)           (None, 21, 21, 240)       921840
_________________________________________________________________
max_pooling2d_5 (MaxPooling2 (None, 10, 10, 240)       0
_________________________________________________________________
conv2d_10 (Conv2D)          (None, 7, 7, 480)         1843680
_________________________________________________________________
conv2d_11 (Conv2D)          (None, 4, 4, 480)         3686880
_________________________________________________________________
global_average_pooling2d_1 ( (None, 480)               0
_________________________________________________________________
dense_1 (Dense)             (None, 600)               288600
_________________________________________________________________
dropout_1 (Dropout)         (None, 600)               0
_________________________________________________________________
dense_2 (Dense)             (None, 400)               240400
_________________________________________________________________
dropout_2 (Dropout)         (None, 400)               0
```

```
_________________________________________________________________
dense_3 (Dense)              (None, 500)               200500
_________________________________________________________________
dropout_3 (Dropout)          (None, 500)               0
_________________________________________________________________
dense_4 (Dense)              (None, 400)               200400
_________________________________________________________________
dropout_4 (Dropout)          (None, 400)               0
_________________________________________________________________
dense_5 (Dense)              (None, 300)               120300
_________________________________________________________________
dropout_5 (Dropout)          (None, 300)               0
_________________________________________________________________
dense_6 (Dense)              (None, 200)               60200
_________________________________________________________________
dropout_6 (Dropout)          (None, 200)               0
_________________________________________________________________
dense_7 (Dense)              (None, 100)               20100
_________________________________________________________________
dense_8 (Dense)              (None, 50)                5050
_________________________________________________________________
dense_9 (Dense)              (None, 2)                 102
=================================================================
Total params: 10,009,652
Trainable params: 10,009,232
Non-trainable params: 420
```

Is $\overline{1}$ - Incision Inferior

```
_________________________________________________________________
Layer (type)                 Output Shape              Param #
=================================================================
conv2d_1 (Conv2D)            (None, 517, 517, 30)      510
_________________________________________________________________
max_pooling2d_1 (MaxPooling2 (None, 258, 258, 30)      0
_________________________________________________________________
conv2d_2 (Conv2D)            (None, 255, 255, 60)      28860
_________________________________________________________________
conv2d_3 (Conv2D)            (None, 252, 252, 90)      86490
_________________________________________________________________
max_pooling2d_2 (MaxPooling2 (None, 126, 126, 90)      0
_________________________________________________________________
conv2d_4 (Conv2D)            (None, 123, 123, 120)     172920
_________________________________________________________________
conv2d_5 (Conv2D)            (None, 120, 120, 150)     288150
```

max_pooling2d_3 (MaxPooling2	(None, 60, 60, 150)	0
conv2d_6 (Conv2D)	(None, 57, 57, 180)	432180
conv2d_7 (Conv2D)	(None, 54, 54, 210)	605010
batch_normalization_1 (Batch	(None, 54, 54, 210)	840
max_pooling2d_4 (MaxPooling2	(None, 27, 27, 210)	0
conv2d_8 (Conv2D)	(None, 24, 24, 240)	806640
conv2d_9 (Conv2D)	(None, 21, 21, 240)	921840
max_pooling2d_5 (MaxPooling2	(None, 10, 10, 240)	0
conv2d_10 (Conv2D)	(None, 7, 7, 480)	1843680
conv2d_11 (Conv2D)	(None, 4, 4, 480)	3686880
global_average_pooling2d_1 (	(None, 480)	0
dense_1 (Dense)	(None, 600)	288600
dropout_1 (Dropout)	(None, 600)	0
dense_2 (Dense)	(None, 500)	300500
dropout_2 (Dropout)	(None, 500)	0
dense_3 (Dense)	(None, 500)	250500
dropout_3 (Dropout)	(None, 500)	0
dense_4 (Dense)	(None, 400)	200400
dropout_4 (Dropout)	(None, 400)	0
dense_5 (Dense)	(None, 300)	120300
dropout_5 (Dropout)	(None, 300)	0
dense_6 (Dense)	(None, 200)	60200

```
_________________________________________________________________
dropout_6 (Dropout)          (None, 200)               0
_________________________________________________________________
dense_7 (Dense)              (None, 100)               20100
_________________________________________________________________
dense_8 (Dense)              (None, 50)                5050
_________________________________________________________________
dense_9 (Dense)              (None, 2)                 102
=================================================================
Total params: 10,119,752
Trainable params: 10,119,332
Non-trainable params: 420
```

N- Nasion

```
_________________________________________________________________
Layer (type)                 Output Shape              Param #
=================================================================
conv2d_1 (Conv2D)            (None, 517, 517, 30)      510
_________________________________________________________________
max_pooling2d_1 (MaxPooling2 (None, 258, 258, 30)      0
_________________________________________________________________
conv2d_2 (Conv2D)            (None, 255, 255, 60)      28860
_________________________________________________________________
conv2d_3 (Conv2D)            (None, 252, 252, 90)      86490
_________________________________________________________________
max_pooling2d_2 (MaxPooling2 (None, 126, 126, 90)      0
_________________________________________________________________
conv2d_4 (Conv2D)            (None, 123, 123, 120)     172920
_________________________________________________________________
conv2d_5 (Conv2D)            (None, 120, 120, 150)     288150
_________________________________________________________________
max_pooling2d_3 (MaxPooling2 (None, 60, 60, 150)       0
_________________________________________________________________
conv2d_6 (Conv2D)            (None, 57, 57, 180)       432180
_________________________________________________________________
conv2d_7 (Conv2D)            (None, 54, 54, 210)       605010
_________________________________________________________________
batch_normalization_1 (Batch (None, 54, 54, 210)       840
_________________________________________________________________
max_pooling2d_4 (MaxPooling2 (None, 27, 27, 210)       0
_________________________________________________________________
conv2d_8 (Conv2D)            (None, 24, 24, 240)       806640
_________________________________________________________________
conv2d_9 (Conv2D)            (None, 21, 21, 240)       921840
```

```
_________________________________________________________________
max_pooling2d_5 (MaxPooling2 (None, 10, 10, 240)      0
_________________________________________________________________
conv2d_10 (Conv2D)           (None, 7, 7, 480)        1843680
_________________________________________________________________
conv2d_11 (Conv2D)           (None, 4, 4, 480)        3686880
_________________________________________________________________
global_average_pooling2d_1 ( (None, 480)              0
_________________________________________________________________
dense_1 (Dense)              (None, 600)              288600
_________________________________________________________________
leaky_re_lu_1 (LeakyReLU)    (None, 600)              0
_________________________________________________________________
dropout_1 (Dropout)          (None, 600)              0
_________________________________________________________________
dense_2 (Dense)              (None, 400)              240400
_________________________________________________________________
dropout_2 (Dropout)          (None, 400)              0
_________________________________________________________________
dense_3 (Dense)              (None, 500)              200500
_________________________________________________________________
dropout_3 (Dropout)          (None, 500)              0
_________________________________________________________________
dense_4 (Dense)              (None, 400)              200400
_________________________________________________________________
dropout_4 (Dropout)          (None, 400)              0
_________________________________________________________________
dense_5 (Dense)              (None, 300)              120300
_________________________________________________________________
dropout_5 (Dropout)          (None, 300)              0
_________________________________________________________________
dense_6 (Dense)              (None, 200)              60200
_________________________________________________________________
dropout_6 (Dropout)          (None, 200)              0
_________________________________________________________________
dense_7 (Dense)              (None, 100)              20100
_________________________________________________________________
dense_8 (Dense)              (None, 50)               5050
_________________________________________________________________
dense_9 (Dense)              (None, 2)                102
=================================================================
Total params: 10,009,652
Trainable params: 10,009,232
Non-trainable params: 420
```

Pog – Pogonion

Layer (type)	Output Shape	Param #
conv2d_1 (Conv2D)	(None, 517, 517, 40)	680
max_pooling2d_1 (MaxPooling2	(None, 258, 258, 40)	0
conv2d_2 (Conv2D)	(None, 255, 255, 80)	51280
conv2d_3 (Conv2D)	(None, 252, 252, 120)	153720
max_pooling2d_2 (MaxPooling2	(None, 126, 126, 120)	0
conv2d_4 (Conv2D)	(None, 123, 123, 160)	307360
conv2d_5 (Conv2D)	(None, 120, 120, 200)	512200
max_pooling2d_3 (MaxPooling2	(None, 60, 60, 200)	0
conv2d_6 (Conv2D)	(None, 57, 57, 240)	768240
conv2d_7 (Conv2D)	(None, 54, 54, 280)	1075480
batch_normalization_1 (Batch	(None, 54, 54, 280)	1120
max_pooling2d_4 (MaxPooling2	(None, 27, 27, 280)	0
conv2d_8 (Conv2D)	(None, 24, 24, 320)	1433920
conv2d_9 (Conv2D)	(None, 21, 21, 320)	1638720
max_pooling2d_5 (MaxPooling2	(None, 10, 10, 320)	0
conv2d_10 (Conv2D)	(None, 7, 7, 640)	3277440
conv2d_11 (Conv2D)	(None, 4, 4, 640)	6554240
global_average_pooling2d_1 (	(None, 640)	0
dense_1 (Dense)	(None, 600)	384600
dropout_1 (Dropout)	(None, 600)	0

```
dense_2 (Dense)           (None, 500)           300500
_________________________________________________________________
dropout_2 (Dropout)       (None, 500)           0
_________________________________________________________________
dense_3 (Dense)           (None, 500)           250500
_________________________________________________________________
dropout_3 (Dropout)       (None, 500)           0
_________________________________________________________________
dense_4 (Dense)           (None, 400)           200400
_________________________________________________________________
dropout_4 (Dropout)       (None, 400)           0
_________________________________________________________________
dense_5 (Dense)           (None, 300)           120300
_________________________________________________________________
dropout_5 (Dropout)       (None, 300)           0
_________________________________________________________________
dense_6 (Dense)           (None, 200)           60200
_________________________________________________________________
dropout_6 (Dropout)       (None, 200)           0
_________________________________________________________________
dense_7 (Dense)           (None, 100)           20100
_________________________________________________________________
dense_8 (Dense)           (None, 50)            5050
_________________________________________________________________
dense_9 (Dense)           (None, 2)             102
=================================================================
Total params: 17,116,152
Trainable params: 17,115,592
Non-trainable params: 560
```

PNS - Posterior nasal spine

```
_________________________________________________________________
Layer (type)              Output Shape          Param #
=================================================================
conv2d_1 (Conv2D)         (None, 517, 517, 30)  510
_________________________________________________________________
max_pooling2d_1 (MaxPooling2 (None, 258, 258, 30)  0
_________________________________________________________________
conv2d_2 (Conv2D)         (None, 255, 255, 60)  28860
_________________________________________________________________
conv2d_3 (Conv2D)         (None, 252, 252, 90)  86490
_________________________________________________________________
max_pooling2d_2 (MaxPooling2 (None, 126, 126, 90)  0
_________________________________________________________________
```

conv2d_4 (Conv2D)	(None, 123, 123, 120)	172920
conv2d_5 (Conv2D)	(None, 120, 120, 150)	288150
max_pooling2d_3 (MaxPooling2	(None, 60, 60, 150)	0
conv2d_6 (Conv2D)	(None, 57, 57, 180)	432180
conv2d_7 (Conv2D)	(None, 54, 54, 210)	605010
batch_normalization_1 (Batch	(None, 54, 54, 210)	840
max_pooling2d_4 (MaxPooling2	(None, 27, 27, 210)	0
conv2d_8 (Conv2D)	(None, 24, 24, 240)	806640
conv2d_9 (Conv2D)	(None, 21, 21, 240)	921840
max_pooling2d_5 (MaxPooling2	(None, 10, 10, 240)	0
conv2d_10 (Conv2D)	(None, 7, 7, 480)	1843680
conv2d_11 (Conv2D)	(None, 4, 4, 480)	3686880
global_average_pooling2d_1 (	(None, 480)	0
dense_1 (Dense)	(None, 600)	288600
dropout_1 (Dropout)	(None, 600)	0
dense_2 (Dense)	(None, 500)	300500
dropout_2 (Dropout)	(None, 500)	0
dense_3 (Dense)	(None, 500)	250500
dropout_3 (Dropout)	(None, 500)	0
dense_4 (Dense)	(None, 400)	200400
dropout_4 (Dropout)	(None, 400)	0
dense_5 (Dense)	(None, 300)	120300

```
dropout_5 (Dropout)        (None, 300)           0
_________________________________________________________________
dense_6 (Dense)            (None, 200)           60200
_________________________________________________________________
dropout_6 (Dropout)        (None, 200)           0
_________________________________________________________________
dense_7 (Dense)            (None, 100)           20100
_________________________________________________________________
dense_8 (Dense)            (None, 50)            5050
_________________________________________________________________
dense_9 (Dense)            (None, 2)             102
=================================================================
Total params: 10,119,752
Trainable params: 10,119,332
Non-trainable params: 420
```

S – Sella

```
_________________________________________________________________
Layer (type)              Output Shape             Param #
=================================================================
conv2d_1 (Conv2D)          (None, 517, 517, 30)     510
_________________________________________________________________
max_pooling2d_1 (MaxPooling2 (None, 258, 258, 30)     0
_________________________________________________________________
conv2d_2 (Conv2D)          (None, 255, 255, 60)     28860
_________________________________________________________________
conv2d_3 (Conv2D)          (None, 252, 252, 90)     86490
_________________________________________________________________
max_pooling2d_2 (MaxPooling2 (None, 126, 126, 90)     0
_________________________________________________________________
conv2d_4 (Conv2D)          (None, 123, 123, 120)    172920
_________________________________________________________________
conv2d_5 (Conv2D)          (None, 120, 120, 150)    288150
_________________________________________________________________
max_pooling2d_3 (MaxPooling2 (None, 60, 60, 150)      0
_________________________________________________________________
conv2d_6 (Conv2D)          (None, 57, 57, 180)      432180
_________________________________________________________________
conv2d_7 (Conv2D)          (None, 54, 54, 210)      605010
_________________________________________________________________
batch_normalization_1 (Batch (None, 54, 54, 210)      840
_________________________________________________________________
max_pooling2d_4 (MaxPooling2 (None, 27, 27, 210)      0
_________________________________________________________________
```

conv2d_8 (Conv2D)	(None, 24, 24, 240)	806640
conv2d_9 (Conv2D)	(None, 21, 21, 240)	921840
max_pooling2d_5 (MaxPooling2	(None, 10, 10, 240)	0
conv2d_10 (Conv2D)	(None, 7, 7, 480)	1843680
conv2d_11 (Conv2D)	(None, 4, 4, 480)	3686880
global_average_pooling2d_1 (	(None, 480)	0
dense_1 (Dense)	(None, 600)	288600
dropout_1 (Dropout)	(None, 600)	0
dense_2 (Dense)	(None, 400)	240400
dropout_2 (Dropout)	(None, 400)	0
dense_3 (Dense)	(None, 500)	200500
dropout_3 (Dropout)	(None, 500)	0
dense_4 (Dense)	(None, 400)	200400
dropout_4 (Dropout)	(None, 400)	0
dense_5 (Dense)	(None, 300)	120300
dropout_5 (Dropout)	(None, 300)	0
dense_6 (Dense)	(None, 200)	60200
dropout_6 (Dropout)	(None, 200)	0
dense_7 (Dense)	(None, 100)	20100
dense_8 (Dense)	(None, 50)	5050
dense_9 (Dense)	(None, 2)	102

Total params: 10,009,652

Trainable params: 10,009,232

Non-trainable params: 420

T1 - Gonion superiorus

```
_________________________________________________________________
Layer (type)                 Output Shape              Param #
=================================================================
conv2d_1 (Conv2D)            (None, 517, 517, 30)      510
_________________________________________________________________
max_pooling2d_1 (MaxPooling2 (None, 258, 258, 30)      0
_________________________________________________________________
conv2d_2 (Conv2D)            (None, 255, 255, 60)      28860
_________________________________________________________________
conv2d_3 (Conv2D)            (None, 252, 252, 90)      86490
_________________________________________________________________
max_pooling2d_2 (MaxPooling2 (None, 126, 126, 90)      0
_________________________________________________________________
conv2d_4 (Conv2D)            (None, 123, 123, 120)     172920
_________________________________________________________________
conv2d_5 (Conv2D)            (None, 120, 120, 150)     288150
_________________________________________________________________
max_pooling2d_3 (MaxPooling2 (None, 60, 60, 150)       0
_________________________________________________________________
conv2d_6 (Conv2D)            (None, 57, 57, 180)       432180
_________________________________________________________________
conv2d_7 (Conv2D)            (None, 54, 54, 210)       605010
_________________________________________________________________
batch_normalization_1 (Batch (None, 54, 54, 210)       840
_________________________________________________________________
max_pooling2d_4 (MaxPooling2 (None, 27, 27, 210)       0
_________________________________________________________________
conv2d_8 (Conv2D)            (None, 24, 24, 240)       806640
_________________________________________________________________
conv2d_9 (Conv2D)            (None, 21, 21, 240)       921840
_________________________________________________________________
max_pooling2d_5 (MaxPooling2 (None, 10, 10, 240)       0
_________________________________________________________________
conv2d_10 (Conv2D)           (None, 7, 7, 480)         1843680
_________________________________________________________________
conv2d_11 (Conv2D)           (None, 4, 4, 480)         3686880
_________________________________________________________________
global_average_pooling2d_1 ( (None, 480)               0
_________________________________________________________________
dense_1 (Dense)              (None, 600)               288600
_________________________________________________________________
```

```
leaky_re_lu_1 (LeakyReLU)    (None, 600)             0
_________________________________________________________________
dropout_1 (Dropout)          (None, 600)             0
_________________________________________________________________
dense_2 (Dense)              (None, 400)             240400
_________________________________________________________________
leaky_re_lu_2 (LeakyReLU)    (None, 400)             0
_________________________________________________________________
dropout_2 (Dropout)          (None, 400)             0
_________________________________________________________________
leaky_re_lu_3 (LeakyReLU)    (None, 400)             0
_________________________________________________________________
dropout_3 (Dropout)          (None, 400)             0
_________________________________________________________________
dense_3 (Dense)              (None, 400)             160400
_________________________________________________________________
dropout_4 (Dropout)          (None, 400)             0
_________________________________________________________________
dense_4 (Dense)              (None, 300)             120300
_________________________________________________________________
dropout_5 (Dropout)          (None, 300)             0
_________________________________________________________________
dense_5 (Dense)              (None, 200)             60200
_________________________________________________________________
dropout_6 (Dropout)          (None, 200)             0
_________________________________________________________________
dense_6 (Dense)              (None, 100)             20100
_________________________________________________________________
dense_7 (Dense)              (None, 50)              5050
_________________________________________________________________
dense_8 (Dense)              (None, 2)               102
=================================================================
Total params: 9,769,152
Trainable params: 9,768,732
Non-trainable params: 420
```

T2 - Gonion inferius

```
_________________________________________________________________
Layer (type)                 Output Shape              Param #
=================================================================
conv2d_1 (Conv2D)            (None, 517, 517, 30)      510
_________________________________________________________________
max_pooling2d_1 (MaxPooling2 (None, 258, 258, 30)      0
_________________________________________________________________
```

conv2d_2 (Conv2D)	(None, 255, 255, 60)	28860
conv2d_3 (Conv2D)	(None, 252, 252, 90)	86490
max_pooling2d_2 (MaxPooling2	(None, 126, 126, 90)	0
conv2d_4 (Conv2D)	(None, 123, 123, 120)	172920
conv2d_5 (Conv2D)	(None, 120, 120, 150)	288150
max_pooling2d_3 (MaxPooling2	(None, 60, 60, 150)	0
conv2d_6 (Conv2D)	(None, 57, 57, 180)	432180
conv2d_7 (Conv2D)	(None, 54, 54, 210)	605010
batch_normalization_1 (Batch	(None, 54, 54, 210)	840
max_pooling2d_4 (MaxPooling2	(None, 27, 27, 210)	0
conv2d_8 (Conv2D)	(None, 24, 24, 240)	806640
conv2d_9 (Conv2D)	(None, 21, 21, 240)	921840
max_pooling2d_5 (MaxPooling2	(None, 10, 10, 240)	0
conv2d_10 (Conv2D)	(None, 7, 7, 480)	1843680
conv2d_11 (Conv2D)	(None, 4, 4, 480)	3686880
global_average_pooling2d_1 (	(None, 480)	0
dense_1 (Dense)	(None, 600)	288600
leaky_re_lu_1 (LeakyReLU)	(None, 600)	0
dropout_1 (Dropout)	(None, 600)	0
dense_2 (Dense)	(None, 400)	240400
leaky_re_lu_2 (LeakyReLU)	(None, 400)	0
dropout_2 (Dropout)	(None, 400)	0

```
leaky_re_lu_3 (LeakyReLU)    (None, 400)            0
_________________________________________________________________
dropout_3 (Dropout)         (None, 400)            0
_________________________________________________________________
dense_3 (Dense)             (None, 400)            160400
_________________________________________________________________
dropout_4 (Dropout)         (None, 400)            0
_________________________________________________________________
dense_4 (Dense)             (None, 300)            120300
_________________________________________________________________
dropout_5 (Dropout)         (None, 300)            0
_________________________________________________________________
dense_5 (Dense)             (None, 200)            60200
_________________________________________________________________
dropout_6 (Dropout)         (None, 200)            0
_________________________________________________________________
dense_6 (Dense)             (None, 100)            20100
_________________________________________________________________
dense_7 (Dense)             (None, 50)             5050
_________________________________________________________________
dense_8 (Dense)             (None, 2)              102
=================================================================
Total params: 9,769,152
Trainable params: 9,768,732
Non-trainable params: 420
```
